# Mitochondrial Dysfunction‐Evoked DHODH Acetylation is Involved in Renal Cell Ferroptosis during Cisplatin‐Induced Acute Kidney Injury

**DOI:** 10.1002/advs.202404753

**Published:** 2024-09-20

**Authors:** Nan‐Nan Liang, Yue‐Yue Guo, Xiao‐Yi Zhang, Ya‐Hui Ren, Yi‐Zhang He, Zhi‐Bing Liu, De‐Xiang Xu, Shen Xu

**Affiliations:** ^1^ Department of Toxicology Anhui Medical University Hefei China 230032; ^2^ Department of Urology the Second Affiliated Hospital of Anhui Medical University Hefei China 230601; ^3^ Department of Blood Transfusion Second Affiliated Hospital of Anhui Medical University Hefei Anhui 230601 China

**Keywords:** acute kidney injury, cisplatin, DHODH acetylation, ferroptosis, SIRT3 SUMOylation

## Abstract

Several studies have observed renal cell ferroptosis during cisplatin‐induced acute kidney injury (AKI). However, the mechanism is not completely clear. In this study, oxidized arachidonic acid (AA) metabolites are increased in cisplatin‐treated HK‐2 cells. Targeted metabolomics showed that the end product of pyrimidine biosynthesis is decreased and the initiating substrate of pyrimidine biosynthesis is increased in cisplatin‐treated mouse kidneys. Mitochondrial DHODH, a key enzyme for pyrimidine synthesis, and its downstream product CoQH_2_, are downregulated. DHODH overexpression attenuated but DHODH silence exacerbated cisplatin‐induced CoQH_2_ depletion and lipid peroxidation. Mechanistically, renal DHODH acetylation is elevated in cisplatin‐exposed mice. Mitochondrial SIRT3 is reduced in cisplatin‐treated mouse kidneys and HK‐2 cells. Both in vitro SIRT3 overexpression and in vivo NMN supplementation attenuated cisplatin‐induced mitochondrial DHODH acetylation and renal cell ferroptosis. By contrast, *Sirt3* knockout aggravated cisplatin‐induced mitochondrial DHODH acetylation and renal cell ferroptosis, which can not be attenuated by NMN. Additional experiments showed that cisplatin caused mitochondrial dysfunction and SIRT3 SUMOylation. Pretreatment with mitochondria‐target antioxidant MitoQ alleviated cisplatin‐caused mitochondrial dysfunction, SIRT3 SUMOylation, and DHODH acetylation. MitoQ pretreatment protected against cisplatin‐caused AKI and renal cell ferroptosis. Taken together, these results suggest that mitochondrial dysfunction‐evoked DHODH acetylation partially contributes to renal cell ferroptosis during cisplatin‐induced AKI.

## Graphical abstract

In this study, it is demonstrated that the downregulation of mitochondrial DHODH, a novel defense mechanism, is involved in renal cell ferroptosis during cisplatin‐induced AKI. The results provide evidence that mitochondrial reactive oxygen species (ROS)‐evoked SIRT3 SUMOylation contributes to cisplatin‐induced DHODH acetylation and mitochondrial DHODH downregulation. Supplementation with mitoquinone (MitoQ), a mitochondria‐targeted antioxidant, and NMN, a precursor for NAD^+^, could efficiently prevent cisplatin‐induced renal cell ferroptosis and AKI.

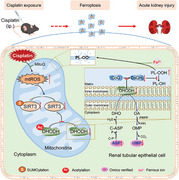

## 1 Introduction

It is well known that rapid reduction of renal function is characteristic of acute kidney injury (AKI).^[^ ^1^, ^2^ ^]^ Accumulating data have demonstrated that drug nephrotoxicity is one of the most common reasons, accounting for 19% of all AKI cases.^[^ ^3^ ^]^ Cisplatin is an anti‐tumor drug that is frequently used to treat solid malignant tumors. The use of cisplatin is limited by dose‐dependent nephrotoxicity.^[^ ^4^, ^5^, ^6^ ^]^ Numerous studies have demonstrated that cisplatin is one of the major causes of clinical AKI.^[^ ^7^, ^8^, ^9^, ^10^ ^]^ Several reports have suggested that ferroptosis, a form of regulated cell death,^11^ ^]^ is involved in cisplatin‐induced AKI.^[^ ^7^, ^12^, ^13^ ^]^ Nevertheless, the exact mechanism for cisplatin‐induced renal cell ferroptosis remains unclear.

The accumulation of oxidized lipids on cellular membranes is the characteristic of ferroptosis.^[^ ^14^ ^]^ Ferroptosis is mainly triggered by accumulation of peroxidation of polyunsaturated fatty acids (PUFA)‐containing phospholipids (PUFA‐PLs).^[^ ^15^, ^16^, ^17^ ^]^ Long chain family member 4 (ACSL4), and lysophosphatidylcholine acyltransferase 3 (LPCAT3) are the vital regulator of PUFA‐PL synthesis.^[^ ^16^ ^]^ ACSL4 catalyzes free PUFAs, such as arachidonic acid (AA), and adrenic acids, with CoA to produce PUFA‐CoAs.^[^ ^15^ ^]^ LPCAT3 re‐esterifies and incorporates PUFA‐CoAs into PUFA‐PLs.^[^ ^18^ ^]^ It is accepted that activation of ACSL4 or LPCAT3 evokes ferroptosis by inducing lipid peroxides on cellular membranes.^[^ ^19^ ^]^ Free ferrous promotes ferroprosis by initiating the Fenton reaction and generating HO^−^ that can directly oxidate PUFA‐PLs.^[^ ^20^ ^]^ Besides, ferrous acts as an essential cofactor for antioxidant enzymes, such as ALOX and POR, participating in lipid peroxidation.^[^ ^21^ ^]^ On the other hand, several ferroptosis defense mechanisms, including X**_c_** ^−^/glutathione peroxidase 4 (GPX4) and ferroptosis suppressor protein 1 (FSP1)/ubiquinol (CoQ10) systems, have been uncovered.^[^ ^21^, ^22^, ^23^ ^]^ System X**_c_** ^−^ regulates the uptake of cysteine, the raw material of reduced glutathione (GSH).^[^ ^24^ ^]^ GPX4 prevents ferroptosis by converting oxidized PUFA‐PLs to PL alcohols in a reduced glutathione (GSH)‐dependent manner.^[^ ^25^ ^]^ FSP1, previously known as apoptosis‐inducing factor mitochondrial 2 (AIFM2), reduces coenzyme Q10 (CoQ) and then inhibits lipid peroxides.^[^ ^22^ ^]^

In this study, we demonstrate that the downregulation of mitochondrial DHODH, a novel defense mechanism,^[^ ^26^ ^]^ is involved in renal cell ferroptosis during cisplatin‐induced AKI. Our results provide evidence that mitochondrial reactive oxygen species (ROS)‐evoked SIRT3 SUMOylation contributes to cisplatin‐induced DHODH acetylation and mitochondrial DHODH downregulation. Supplementation with mitoquinone (MitoQ), a mitochondria‐targeted antioxidant, and NMN, a precursor for NAD^+^, could efficiently prevent cisplatin‐induced renal cell ferroptosis and AKI.

## 2 Results

### 2.1 Cisplatin‐Induced Acute Kidney Injury is Accompanied by Renal Cell Ferroptosis

**Figure 1:**
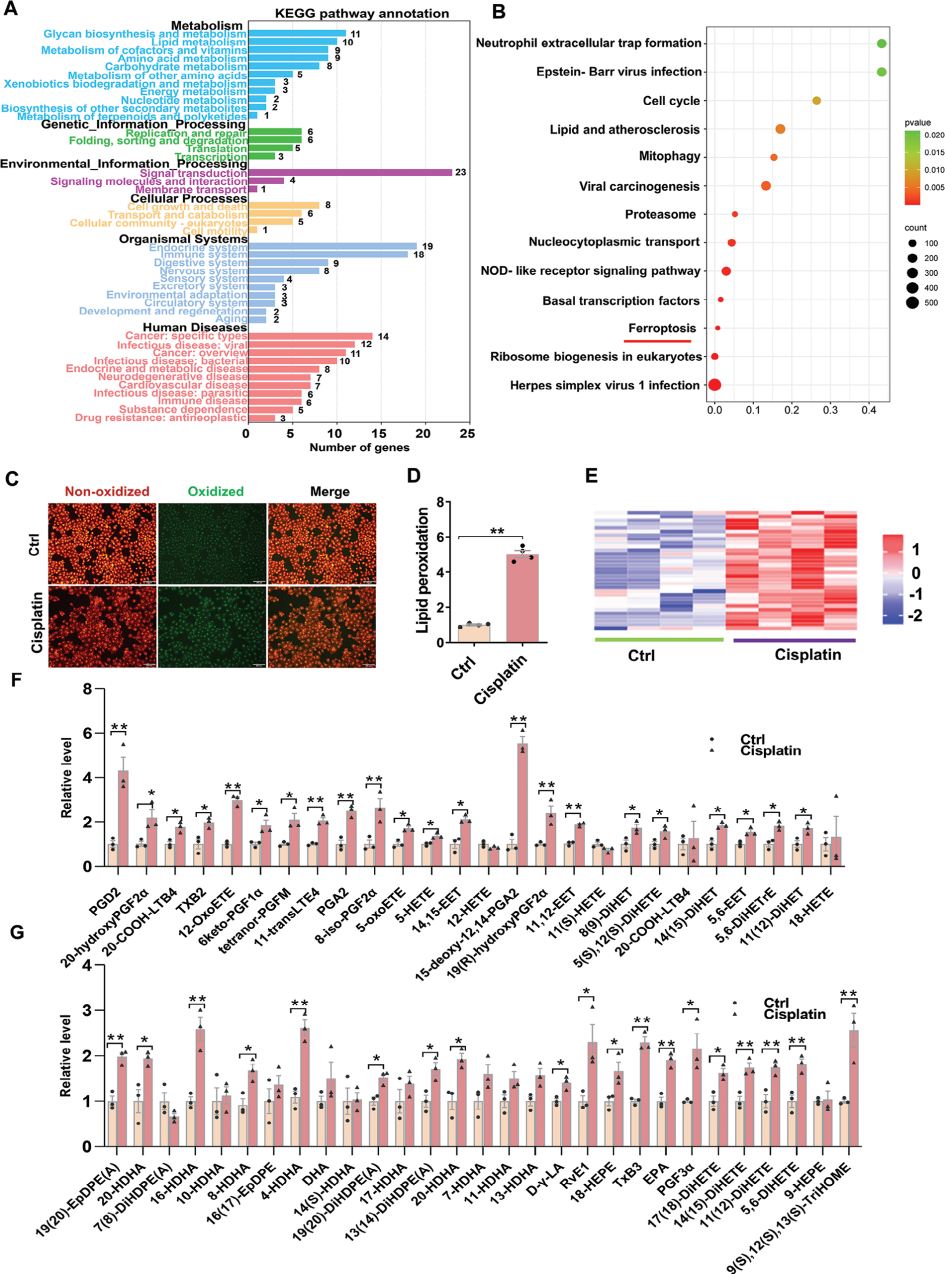
Cisplatin‐induced acute kidney injury and renal cell ferroptosis. HK‐2 cells were incubated with cisplatin (10 µmol L^−1^) for 72 h. (A) The KEGG pathway enrichment analysis was shown. N = 3. (B) Top of KEGG pathway enrichment analysis. (C‐D) C11‐BODIPY^581/591^ was used to evaluate lipid peroxidation. Experiment was repeated two times, N = 4. (C) Representative pictures: Scale bar = 200 µm. (D) Quantitative analysis of oxidized lipids. E‐G) Targeted oxidized lipids metabolomics were evaluated using LC‐MS/MS. Experiment was repeated two times, N = 3. (E) Heatmap of targeted oxidized lipids metabolomics. (F) Oxidized metabolites of AA. (G) Oxidized metabolites of DHA, EPA, GLA, and LA metabolism. Quantitative data were shown as data pots and S.E.M. *p < 0.05, **p < 0.01.

First, cisplatin‐induced pathological changes were analyzed in renal cortex. Cisplatin induced tubule expansion, and tubular epithelium vacuolization in mouse kidneys (Figure S1A,B, Supporting Information). Serum Scr and BUN were evaluated (Figure S1C,D, Supporting Information). As expected, serum Scr and BUN were increased 72 h after cisplatin. Renal 4‐HNE, a lipid peroxidation marker, was detected by IHC. Renal 4‐HNE^+^ area was detected, beginning at 24 h and remaining increased at 72 h after cisplatin (Figure S1E,F, Supporting Information). Besides, mitochondrial morphological was observed using TEM. As shown in Figure S1G‐J (Supporting Information), mitochondrial area, form factor, and mitochondrial perimeter were reduced in cisplatin‐treated mouse kidneys. Next, RNA‐seq analysis was performed in cisplatin‐treated HK‐2 cells (Figure S1K, Supporting Information). The results showed that the ferroptosis pathway was enriched in cisplatin‐exposed HK‐2 cells (**Figure** **1**A,B). The main characteristic of ferroptosis is lipid peroxidation. The contents of oxidized lipids, as evaluated using immunofluorescence, were increased in cisplatin‐treated HK‐2 cells (Figure 1C,D). Targeted oxidized lipids metabolomics was detected using liquid chromatography‐tandem mass spectrometry (LC‐MS/MS). As shown in Figure 1E, a significant difference in oxidized lipids was observed between cisplatin‐treated HK‐2 cells and controls. Further analysis showed that the contents of oxidized arachidonic acid (AA) metabolites, including 12‐oxoETE, 14,15‐ETE, 5HETE, PGA2, and 5,6‐ETE, were increased in cisplatin‐exposed HK‐2 cells (Figure 1F). Moreover, a significant difference in oxidized docosahexaenoic acid (DHA), gamma‐linolenic acid (GLA), eicosapentaenoic acid (EPA), and linoleic acid (LA) metabolites was shown between cisplatin‐exposed HK‐2 cells and controls (Figure 1G). Finally, the effect of ferrostatin‐1(Fer‐1), a ferroptosis inhibitor, on cisplatin‐induced pathology and renal function was analyzed. As shown in Figure S1L,M (Supporting Information). pretreatment with Fer‐1 alleviated cisplatin‐induced tubule expansion and tubular epithelium vacuolization in mouse kidneys. Pretreatment with Fer‐1 ameliorated cisplatin‐induced elevation of Scr and BUN (Figure S1N,O, Supporting Information).

### 2.2 Mitochondrial DHODH is Downregulated in Cisplatin‐Exposed HK‐2 Cells and Mouse Kidneys

**Figure 2:**
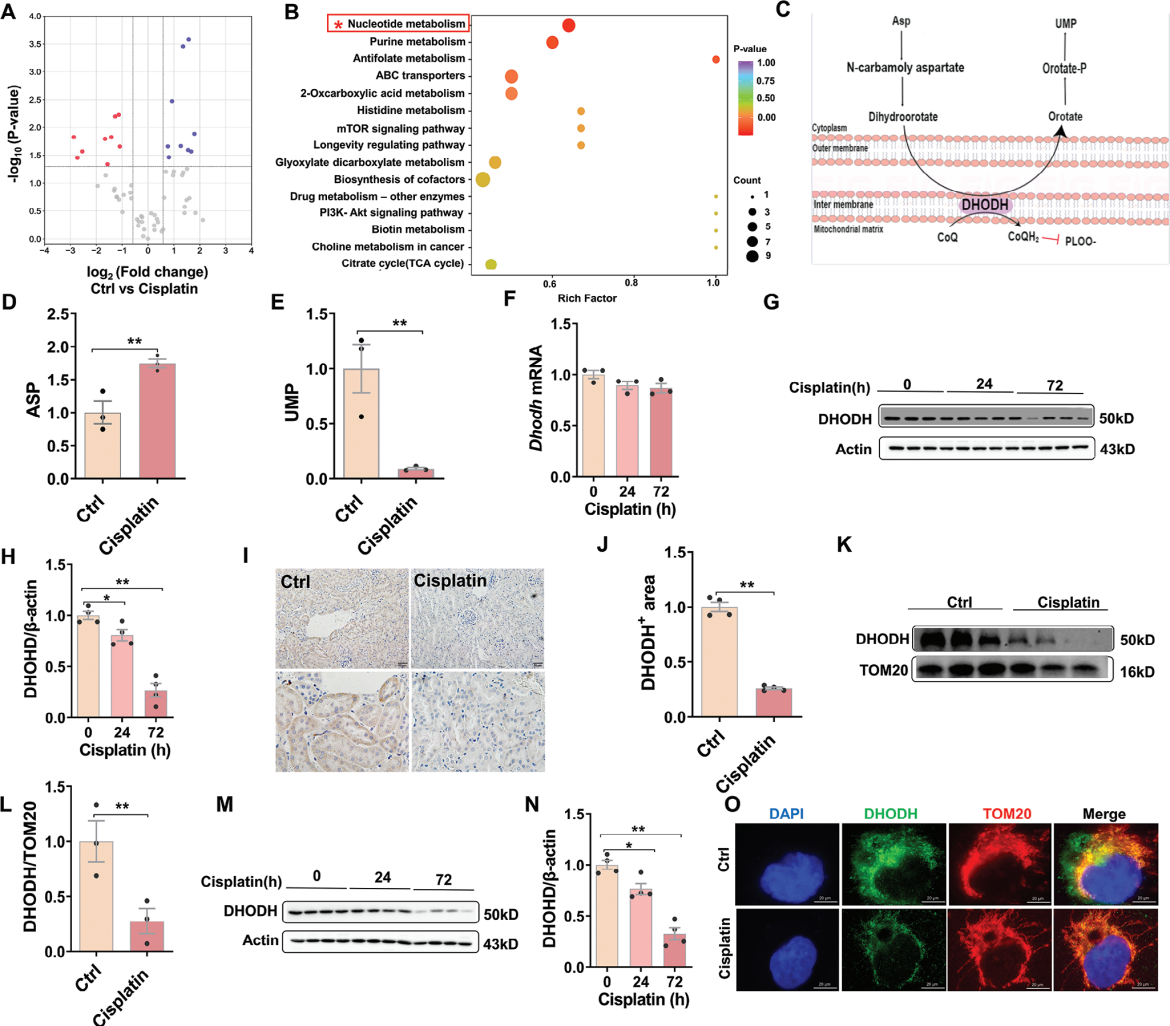
Mitochondrial DHODH in cisplatin‐treated HK‐2 cells and mouse kidneys. A‐M) Adult ICR male mice were exposed with cisplatin (20 mg kg^−1^, intraperitoneal injection). Mouse kidneys were collected at different times 24 and 72 h after cisplatin. A, B targeted metabolomics was analyzed by LC‐MS/MS. N = 3. (A) Volcano plot of targeted metabolomics in mouse kidneys at 72 h after cisplatin treatment. (B) Pathway enrichment analysis of targeted metabolomics. (C) Simplified schematics of de novo pyrimidine biosynthesis pathway. (D) Relative ASP level. (E) Relative UMP level. (F) Real‐time RT‐PCR was used to evaluate *Dhodh* mRNA. Experiment was repeated two times, N = 3. (G‐H) DHODH was analyzed using Western blot. N = 4. (G) Representative pictures. (H) DHODH. IHC was used to analyze renal 4‐HNE. Experiment was repeated two times, N = 4. (I) Representative pictures: Scale bar = 50 µm. (J) Renal 4‐HNE^+^ area. (K) Colocalization of renal DHODH (green signal) with TOM20 (red signal) was determined by immunofluorescence. Nuclei were counterstained with DAPI (blue signal). Scale bar = 100 µm. (L‐M) Mitochondrial DHODH was analyzed by Western blot. Experiment was repeated two times, N = 3. (L) Representative pictures. (M) Mitochondrial DHODH. (N‐O) HK‐2 cells were cultured with cisplatin (10 µmol L^−1^) for either 24 or 72 h. DHODH was measured by Western blot. Experiment was repeated two times, N = 4. (N) Representative pictures. (O) DHODH. (P) Colocalization of DHODH (green signal) with TOM20 (red signal) was determined by immunofluorescence in HK‐2 cells at 72 h after cisplatin treatment. Nuclei were counterstained with DAPI (blue signal). Scale bar = 20 µm. Quantitative data were shown as data pots and S.E.M. *p < 0.05, **p < 0.01.

As show in **Figure** **2**A, targeted cellular metabolites were detected using LC‐MS/MS. Targeted metabolomic analysis showed that nucleotide metabolism pathway was enriched in cisplatin‐treated renal tissues (Figure 2B). As shown in Figure 2C, UMP is an end product of pyrimidine biosynthesis and ASP is an intermediate of pyrimidine biosynthesis. Metabolomic analysis revealed a reduction of UMP and an accumulation of ASP in cisplatin‐treated renal tissues (Figure 2D,E). DHODH, located in the mitochondrial intermembrane, is a crucial enzyme for pyrimidine biosynthesis.^[^ ^27^ ^]^ Despite no significant difference in renal *Dhodh* mRNA (Figure 2F), DHODH protein was reduced in cisplatin‐treated mouse kidneys (Figure 2G,H). IHC showed that renal DHODH^+^ area was reduced (Figure 2I,J). Mitochondrial DHODH protein was further analyzed by immunofluorescence and Western blot. Immunofluorescence showed that the co‐location of mitochondrial DHODH with TOM20 was reduced in cisplatin‐treated mouse kidneys (Figure 2K). As shown in Figure 2L,M, mitochondrial DHODH protein was reduced in cisplatin‐treated mouse kidneys. Finally, DHODH protein was detected in cisplatin‐treated HK‐2 cells. DHODH was decreased in cisplatin‐exposed HK‐2 cells (Figure 2N,O). The co‐location of mitochondrial DHODH with TOM20 was decreased in cisplatin‐exposed HK‐2 cells (Figure 2O). DHODH converts CoQ to CoQH_2_ in de novo pyrimidine synthesis.^[^ ^26^ ^]^ As presented in Figure S2A,B (Supporting Information), CoQ was increased and CoQH_2_ was decreased in cisplatin‐treated HK‐2 cells. The ratio of CoQ to CoQH_2_ was elevated in cisplatin‐exposed HK‐2 cells (Figure S2C, Supporting Information).

### 2.3 Mitochondrial DHODH Downregulation is Involved in Cisplatin‐Induced Renal Cell Ferroptosis

**Figure 3:**
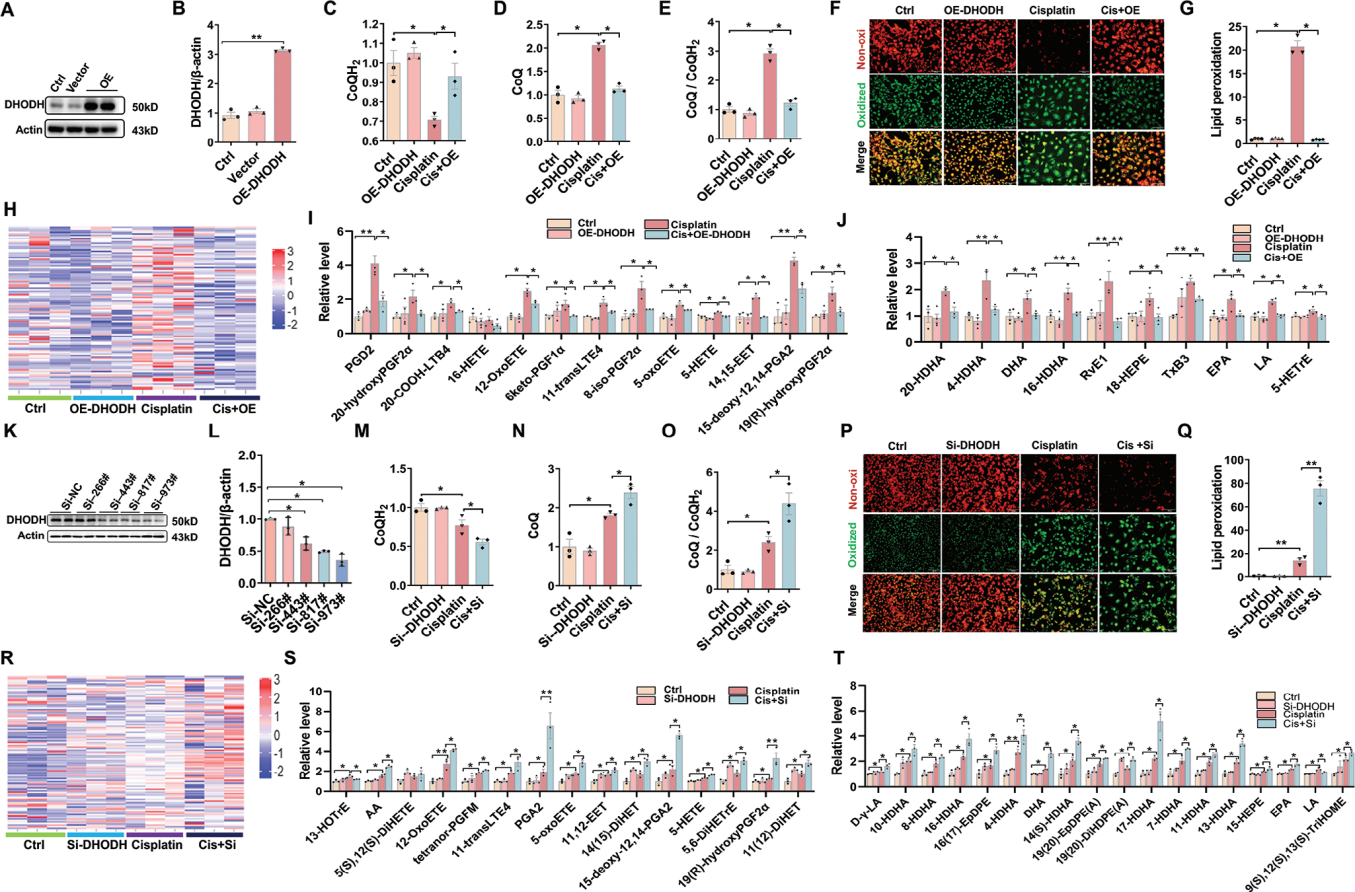
Mitochondrial DHODH downregulation is involved in cisplatin‐induced renal cell ferroptosis. A‐B HK‐2 cells were treated with DHODH plasmids. Experiment was repeated two times, N = 3‐4. A) DHODH was measured by Western blot. (B) Quantitative analysis of DHODH. C‐J) HK‐2 cells were treated with DHODH plasmids before cisplatin (10 µmol L^−1^) treatment. (C) CoQH_2_. (D) CoQ. (E) CoQ/CoQH_2_. (F‐G) C11‐BODIPY^581/591^ was used to evaluate oxidized lipids. Experiment was repeated two times, N = 4. (F) Representative pictures: Scale bar = 100 µm. (G) Quantitative analysis of oxidized lipids. (H‐J) Targeted oxidized lipids metabolomics were determined using LC‐MS/MS. N = 4 (H) Heatmap of targeted oxidized lipids metabolomics. (I) Quantitative analysis of oxidized metabolites of AA. (J) Quantitative analysis of oxidized metabolites of DHA, EPA, GLA, and LA. K, L HK‐2 cells were treated with siRNA specifically targeting DHODH. K) DHODH was measured by Western blot.(L) Quantitative analysis of DHODH. M‐T) HK‐2 cells were treated with siRNA specifically targeting DHODH before cisplatin (10 µmol/L) treatment. (M) CoQH_2_. (N) CoQ. (O) CoQ/CoQH_2_. (P‐Q) C11‐BODIPY^581/591^ was used to evaluate lipid peroxidation. Experiment was repeated two times, N = 3. (P) Representative pictures: Scale bar = 100 µm. (Q) Quantitative analysis of oxidized lipids. (R‐T) Targeted oxidized lipids metabolomics were determined using LC‐MS/MS. N = 3. (R) Heatmap of targeted oxidized lipids metabolomics. (S) Quantitative analysis of oxidized metabolites of AA. (T) Quantitative analysis of oxidized metabolites of DHA, EPA, GLA, and LA. Quantitative data were shown as data pots and S.E.M. *p < 0.05, **p < 0.01.

To verify the role of DHODH downregulation in cisplatin‐induced renal cell ferroptosis, DHODH overexpression was constructed in HK‐2 cells (**Figure** **3**A,B). As expected, CoQH_2_ was reduced in cisplatin‐exposed HK‐2 cells (Figure 3C). By contrast, CoQ content was elevated in cisplatin‐exposed HK‐2 cells (Figure 3D). The ratio of CoQ/CoQH_2_ was elevated in cisplatin‐exposed HK‐2 cells (Figure 3E). Interestingly, cisplatin‐induced reduction of CoQH_2_ content was attenuated in DHODH*^OE^* HK‐2 cells (Figure 3C). Correspondingly, cisplatin‐induced elevation of CoQ content and CoQ/CoQH_2_ was reversed in DHODH*^OE^* HK‐2 cells (Figure 3D,E). The effect of DHODH overexpression on cisplatin‐induced oxidized lipids was evaluated by immunofluorescence. As shown in Figure 3F,G, cisplatin‐induced lipid peroxidation was attenuated in DHODH*^OE^* HK‐2 cells. Targeted lipid peroxidation metabolomics was determined using LC‐MS/MS (Figure 3H). As shown in Figure 3I, DHODH overexpression markedly reversed cisplatin‐induced elevation of the oxidized AA metabolites, such as 12‐oxoETE, 11‐transLTE4, 5‐oxoETE, 5HETE, 14,15‐ETE, and 19(R)‐hydroxyPGF2α. Moreover, DHODH overexpression gene alleviated cisplatin‐induced elevation of oxidized DHA, GLA, EPA, and LA metabolites (Figure 3J). Subsequently, HK‐2 cells were pretreated with DHODH siRNA (Figure 3K,L). As shown in Figure 3M, cisplatin‐induced reduction of CoQH_2_ was exacerbated in DHODH‐silenced HK‐2 cells. Accordingly, cisplatin‐induced elevation of CoQ and CoQ/CoQH_2_ was aggravated in DHODH‐silenced HK‐2 cells (Figure 3N,O). Interestingly, the cisplatin‐caused elevation of oxidized lipids, determined by immunofluorescence, was aggravated in DHODH‐silenced HK‐2 cells (Figure 3P,Q). Finally, Targeted lipid peroxidation metabolomics was detected using LC‐MS/MS (Figure 3R). Cisplatin‐caused elevation of the oxidized AA metabolites, including 12‐oxoETE, 11‐transLTE4, 5‐oxoETE, 5H ETE, 14,15‐DiHET, and 19(R)‐hydroxyPGF2α was exacerbated in DHODH‐silenced HK‐2 cells (Figure 3S). Moreover, cisplatin‐caused elevation of oxidized DHA, GLA, EPA, and LA metabolites was aggravated in DHODH‐silenced HK‐2 cells (Figure 3T).

### 2.4 Cisplatin Induces Mitochondrial SIRT3 Reduction and DHODH Acetylation in HK‐2 Cells and Mouse Kidneys

**Figure 4:**
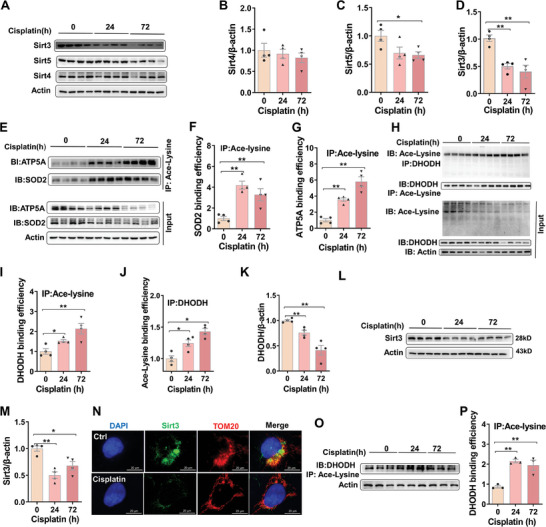
Cisplatin induces mitochondrial SIRT3 reduction and DHODH acetylation in HK‐2 cells and mouse kidneys. (A‐K) Adult ICR male mice were exposed with cisplatin (20 mg kg^−1^, intraperitoneal injection). Mouse kidneys were collected at different times 24 and 72 hours after cisplatin. Experiment was repeated two times, N = 4. (A‐D) SIRT3, SIRT4, and SIRT5 were measured by Western blot. (A) Representative pictures. (B) SIRT4. (C) SIRT5. (D) SIRT3. (E‐G) Co‐IP was used to analyze acetylation of SOD2 and ATP5A. Experiment was repeated two times, N = 4. (E) Representative pictures. (F) Acetylated SOD2. (G) Acetylated ATP5A. (H‐K) Co‐IP was used to analyze acetylation of DHODH. (H) Representative pictures. Experiment was repeated two times, N = 4. (I) Acetylated DHODH by Co‐IP with anti‐ace‐lysine. (J) Acetylated DHODH by Co‐IP with anti‐DHDOH. (K) DHODH. (L‐M) HK‐2 cells were incubated with cisplatin for either 24 or 72 h. Experiment was repeated two times, N = 4. (L) SIRT3 was measured by Western blot. (M) Quantitative analysis of SIRT3. N) Colocalization of SIRT3 (green signal) with TOM20 (red signal) was determined by immunofluorescence in HK‐2 cells at 72 h after cisplatin treatment. Nuclei were counterstained with DAPI (blue signal). (O‐P) Co‐IP was used to analyze acetylation of DHODH. Experiment was repeated two times, N = 3. (O) Representative pictures. (P) Acetylated DHODH by Co‐IP with anti‐ace‐lysine. Quantitative data were shown as data pots and S.E.M. *p < 0.05, **p < 0.01.

First, renal SIRT4, SIRT5, and SIRT3 were detected in cisplatin‐treated mice. No difference on renal SIRT4 was shown between cisplatin‐treated mice and controls (**Figure** **4**A,B). Renal SIRT5 was slightly reduced in cisplatin‐treated mice (Figure 4A,C). Renal SIRT3 was obviously reduced in cisplatin‐treated mice (Figure 4A,D). SIRT3 functions as a deacetylase that regulates mitochondrial proteins.^[^ ^28^ ^]^ Thus, renal acetylated ATP5A and SOD2, two downstream molecules of SIRT3, were then analyzed in cisplatin‐treated mice. As show in Figure 4E–G, renal acetylated ATP5A and SOD2 were elevated in cisplatin‐treated mice. DHODH is located in mitochondrial intermembrane. Renal DHODH acetylation also was analyzed in cisplatin‐treated mice. As shown in Figure 4H,I, renal acetylated DHODH, determined by Co‐IP with anti‐Ace‐lysine, was elevated in cisplatin‐treated mice. Accordingly, renal acetylated DHODH, determined by Co‐IP with anti‐DHODH, was elevated in cisplatin‐treated mice (Figure 4H,J). Renal DHODH protein was reduced in cisplatin‐treated mice. Next, SIRT3 protein was measured in cisplatin‐treated HK‐2 cells. SIRT3 protein was reduced in cisplatin‐treated HK‐2 cells (Figure 4L,M). The co‐location of SIRT3 with TOM20 was observably decreased in cisplatin‐treated HK‐2 cells (Figure 4N). Finally, we analyzed DHODH acetylation in cisplatin‐treated HK‐2 cells. As shown in Figure 4O,P, acetylated DHODH was elevated in cisplatin‐treated HK‐2 cells.

### 2.5 Supplementation with NMN and *Sirt3* Overexpression Alleviates Cisplatin‐Induced Mitochondrial DHODH Acetylation and Renal Cell Ferroptosis

**Figure 5:**
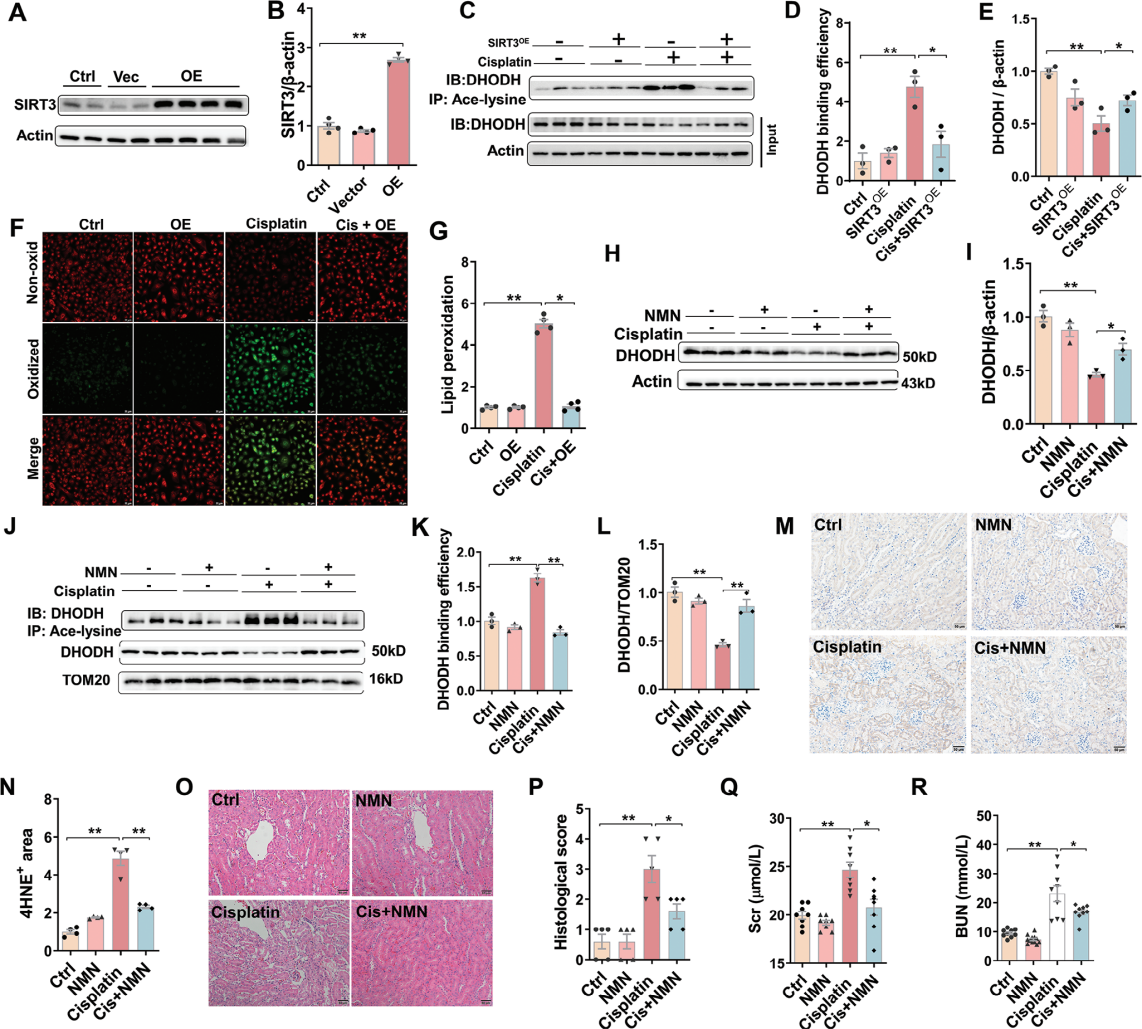
The effect of NMN and *Sirt3* overexpression on cisplatin‐induced mitochondrial DHODH acetylation and renal cell ferroptosis. (A‐G) HK‐2 cells were treated with SIRT3 plasmids. SIRT3 was measured by Western blot at 24 h after SIRT3 plasmids transfection. Experiment was repeated two times. (A) Representative pictures. (B) SIRT3. (C‐E) Co‐IP was used to analyze acetylation of DHODH in HK‐2 cells at 72 h after cisplatin. Experiment was repeated two times, N = 3. (C) Representative pictures. (D) Acetylated DHODH by Co‐IP with anti‐ace‐lysine. (E) DHODH. (F‐G) C11‐BODIPY^581/591^ was used to evaluate oxidized lipids in HK‐2 cells at 24 h after cisplatin. Experiment was repeated two times, N = 4. (F) Representative pictures: Scale bar = 20 µm. (G) Quantitative analysis of oxidized lipids. H‐R) Adult ICR male mice were pretreated with NMN (500 mg kg^−1^) for consecutive five days before cisplatin (20 mg kg^−1^). Mouse kidneys and blood serum were collected 72 h after cisplatin. H, I Western blot was used to evaluate renal DHODH in NMN‐pretreated mice. (H) Representative pictures. (I) DHODH. (J‐K) Co‐IP was used to analyze mitochondrial DHODH acetylation. (J) Representative pictures. (K) Mitochondrial DHODH acetylation. (L) Mitochondrial DHODH. (M‐N) Renal 4‐HNE residues were analyzed using IHC. (M) Representative pictures: Scale bar = 50 µm. (N) Renal 4‐HNE^+^ area was evaluated. (O‐P) H&E staining was used to evaluate renal histopathology. (O) Representative pictures: Scale bar = 50 µm. (P) Histopathological scores. Q‐O Renal function was measured. (**N**) Scr. (**O**) BUN. Quantitative data were shown as data pots and S.E.M. *p < 0.05, **p < 0.01.

SIRT3 functions as an NAD^+^‐dependent deacetylase. To explore the potential role of SIRT3 in cisplatin‐induced DHODH acetylation, SIRT3 overexpression was constructed in HK‐2 cells (**Figure** **5**A,B). As expected, cisplatin‐induced DHODH acetylation was reversed in SIRT3*^OE^* HK‐2 cells (Figure 5C,D). Correspondingly, cisplatin‐induced reduction of DHODH was alleviated in SIRT3^OE^ HK‐2 cells (Figure 5C,E). The effect of SIRT3 overexpression on cisplatin‐induced oxidized lipids was evaluated by immunofluorescence. As shown in Figure 5F,G, cisplatin‐induced lipid peroxidation was attenuated in SIRT3*^OE^* HK‐2 cells. NMN, a main precursor of NAD^+^,^[^ ^29^, ^30^, ^31^ ^]^ was used to activate SIRT3 activity. To confirm the role of SIRT3 in cisplatin‐induced DHODH acetylation in the vivo experiments, mice were pretreatment with NMN. As expected, pretreatment with NMN markedly alleviated cisplatin‐induced reduction of DHODH in mouse kidneys (Figure 5H,I). Moreover, pretreatment with NMN attenuated cisplatin‐induced mitochondrial DHODH acetylation and DHODH reduction (Figure 5J,K). The effect of NMN on cisplatin‐induced renal cell ferroptosis was then analyzed. Cisplatin‐induced elevation of 4‐HNE^+^ area was attenuated in NMN‐pretreated mouse kidneys (Figure 5M,N). The effect of NMN on cisplatin‐induced pathology and renal function injury was then analyzed. The results showed that pretreatment with NMN protected against cisplatin‐induced pathological damage (Figure 5O,P). As shown in Figure 5Q,R, pretreatment with NMN alleviated cisplatin‐induced elevation of Scr and BUN.

### 2.6 *Sirt3* Knockout Aggravates Cisplatin‐Induced DHODH Acetylation and Renal Cell Ferroptosis in Mouse Kidneys

**Figure 6:**
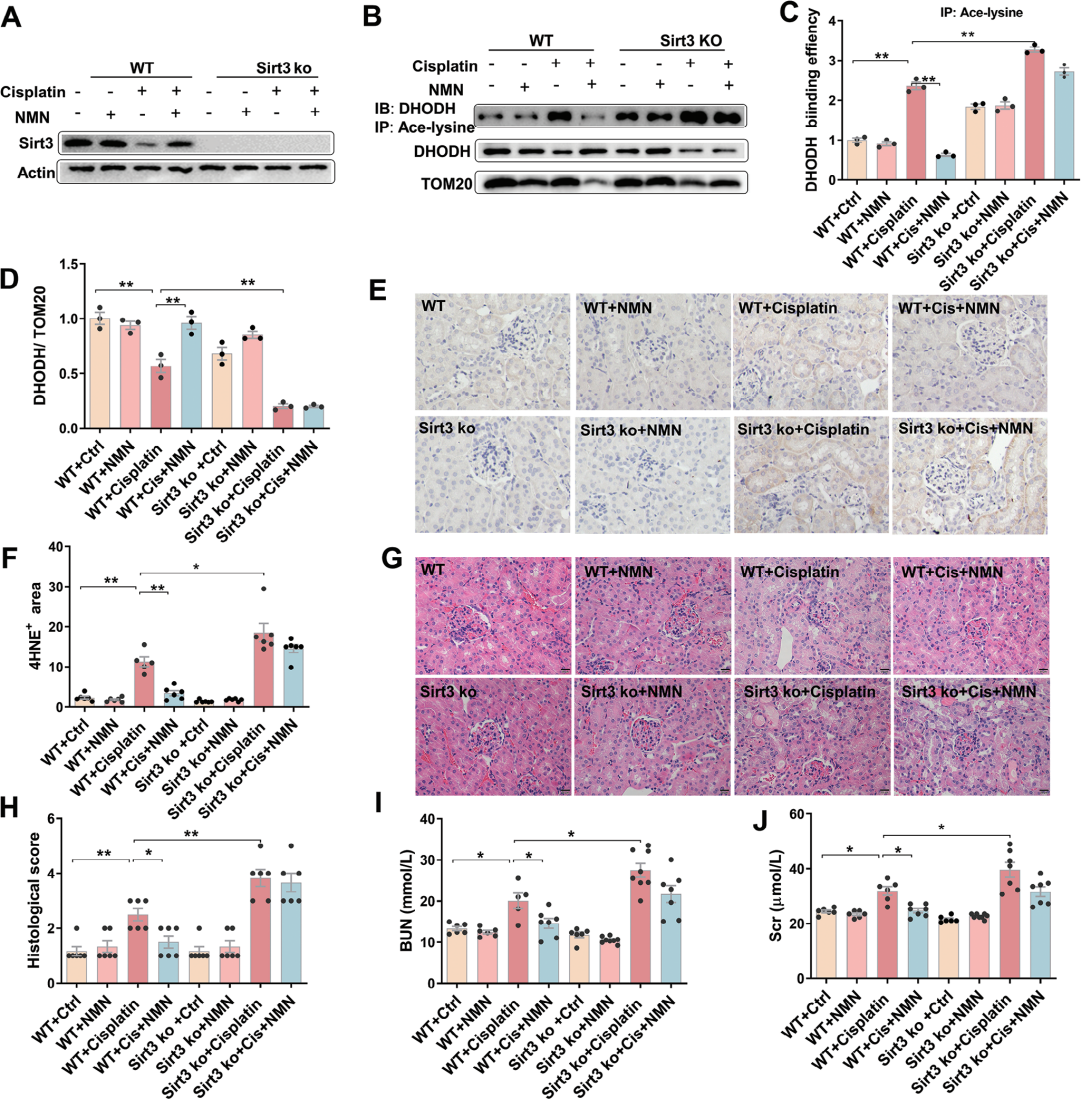
*Sirt3* knockout aggravates cisplatin‐induced DHODH acetylation and renal cell ferroptosis in mouse kidneys. WT and *Sirt3* ^−/−^ mice were pretreated with NMN (500 mg kg^−1^) for consecutive five days before cisplatin (20 mg kg^−1^). Kidney and blood serum were collected 72 h after cisplatin. (A) Western blot was used to evaluate renal SIRT3. (B‐D) Co‐IP was used to analyze mitochondrial DHODH acetylation. The experiment was repeated two times. N = 3. (B) Representative pictures. (C) Mitochondrial DHODH acetylation. (D) Mitochondrial DHODH. (E‐F) IHC was used to analyze renal 4‐HNE. N = 6. (E) Representative pictures: Scale bar = 20 µm. (F) Renal 4‐HNE^+^ area. (G‐H) H&E staining was used to evaluated renal histopathology. (G) Representative pictures: Scale bar = 20 µm. (H) Histopathological scores. Renal function was measured. N = 5 – 8. (I) BUN. (J) Scr. Quantitative data were shown as data pots and S.E.M. *p < 0.05, **p < 0.01.

To further verify whether SIRT3 downregulation is involved in cisplatin‐induced DHODH acetylation, *Sirt3^−/−^* mice were constructed as schematic diagram in Figure S3A (Supporting Information). Subsequently, genotypes were detected to select the *Sirt3^−/‐^* mice (Figure S3B‐D, Supporting Information) and results showed that renal SIRT3 protein was completely deleted in *Sirt3^−/−^* mice (**Figure** **6**A; Figure S3E,F, Supporting Information). Next, acetylated DHODH was analyzed in wide‐type and *Sirt3^−/‐^* mice. As expected, NMN attenuated cisplatin‐induced DHODH acetylation in wide‐type mice (Figure 6B,C). Interestingly, acetylated DHODH was increased in *Sirt3^−/‐^* as compared with wide‐type mice (Figure 6B,C). Moreover, cisplatin‐induced mitochondrial DHODH acetylation was aggravated in *Sirt3^−/‐^* mice, which could not be attenuated by NMN (Figure 6B,C). Correspondingly, cisplatin‐induced reduction of mitochondrial DHODH protein was aggravated in *Sirt3^−/‐^* mice, which could also not be attenuated by NMN (Figure 6B–D). Renal 4‐HNE residue was evaluated by IHC. As shown in Figure 6E,F, cisplatin‐induced elevation of 4‐HNE^+^ area was aggravated in *Sirt3^−/‐^* mice, which could not be rescued by NMN supplementation. Consistent with this, more serious pathological changes and renal dysfunction were observed in cisplatin‐treated *Sirt3^−/‐^* mice, which could also not be alleviated by NMN (Figure 6G–J).

### 2.7 Cisplatin Induces Mitochondrial Dysfunction and SIRT3 SUMOylation in HK‐2 Cells and Mouse Kidneys

**Figure 7:**
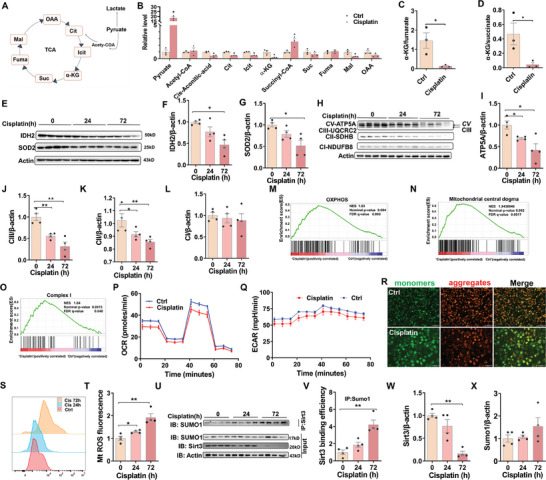
Cisplatin induces mitochondrial dysfunction and SIRT3 SUMOylation.(A) Simplified schematics of the TCA cycle. (B‐L) Adult ICR male mice were exposed with cisplatin (20 mg kg^−1^, intraperitoneal injection). Mouse kidneys were collected at different times 24 and 72 h after cisplatin. (B‐D) TCA cycle metabolites were evaluated by LC‐MS/MS in mouse kidneys at 72 h after cisplatin. N = 3. (B) Relative level of TCA cycle metabolites. (C) α‐KG/fumarate. (D) α‐KG/succinate. (E‐G) Renal IDH2 and SOD2 were measured by Western blot. The experiment was repeated two times. N = 4. (E) Representative pictures. (F) IDH2. (G) SOD2. (H‐L) Four subunits of oxidative phosphorylation were measured by Western blot. Representative pictures and quantitative analysis of four subunits. The experiment was repeated two times. N = 4. (H) Representative pictures. (I) CV‐ATP5A. (J) CIII‐UQCRC2. (K) CII‐SDHB. (L) CI‐NDUFB8. HK‐2 cells were cultured with cisplatin (10 µm L^−1^) for 72 h. Gene set enrichment analysis (GSEA) was presented. (P‐Q) OCR and ECAR were measured by seahorse in HK‐2 cells at 72 h after cisplatin treatment. The experiment was repeated two times. N = 3. (P) OCR. (Q) ECAR. (R) Mitochondrial membrane potential was elevated using JC‐1 in HK‐2 cells at 72 h after cisplatin treatment. Scale bar = 100 µm. (S‐T) HK‐2 cells were cultured with cisplatin (10 µm L^−1^) for 24 h or 72 h. Mitochondrial ROS was evaluated using Flow cytometry. (S) Representative pictures. (T) Mitochondrial ROS. The experiment was repeated two times. N = 4. (U‐X) Adult ICR male mice were exposed with cisplatin (20 mg kg^−1^, intraperitoneal injection). Mouse kidneys were collected at different times 24 and 72 h after cisplatin. The experiment was repeated two times. N = 4. Co‐IP was used to analyze SIRT3 SUMOylation. (U) Representative pictures. (V) SUMOylated SIRT3. (W) SIRT3. X) Sumo1. Quantitative data were shown as data pots and S.E.M. *P < 0.05, **P < 0.01.

To determine the impact of cisplatin on renal mitochondrial function, targeted TCA metabolomics was detected by LC‐MS/MS (**Figure** **7**A–D). Several TCA metabolites, including cis‐aconitic acid, citrate, isocitrate, alpha‐ketoglutarate (α‐KG) and malate, were decreased in cisplatin‐treated mouse kidneys (Figure 7B). Moreover, the ratios of renal α‐KG to fumarate and renal α‐KG to succinate were reduced in cisplatin‐treated mice (Figure 7C,D). Several mitochondrial proteins were then measured. Renal IDH2 and SOD2 were downregulated in cisplatin‐treated mice (Figure 7E,F). Renal CV‐ATP5A, CIII‐UQCRC2, and CII‐SDHB, three subunits of oxidative phosphorylation, were reduced in cisplatin‐treated mice (Figure 7H–L). Next, the impacts of cisplatin on mitochondrial functions were further verified in HK‐2 cells. Transcriptome analysis showed that OXPHOS, mitochondrial central dogma, and complex 1 were enriched in cisplatin‐treated HK‐2 cells (Figure 7M–P). Moreover, Seahorse Cell‐Mito Stress tests revealed that oxygen consumption rate (OCR) and extracellular acidification rate (ECAR) were down‐regulated in cisplatin‐treated HK‐2 cells (Figure 7P,Q). In addition, mitochondrial membrane potential was reduced in cisplatin‐treated HK‐2 cells (Figure 7R). It is known that the deacetylase activity of mitochondrial SIRT3 is downregulated by ROS‐mediated SIRT3 SUMOylation.^[^ ^32^, ^33^, ^34^ ^]^ As shown in Figure 7S,T, mitochondrial ROS were obviously elevated in cisplatin‐exposed HK‐2 cells. Renal SIRT3 SUMOylation was accordingly upregulated in cisplatin‐treated mice (Figure 7U–X).

### 2.8 MitoQ Attenuates Cisplatin‐Induced SIRT3 SUMOylation

**Figure 8:**
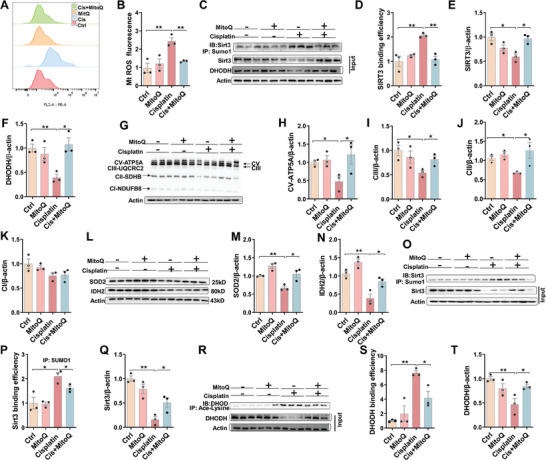
The effect of MitoQ on mitochondrial dysfunction, Sirt3 SUMOylation and DHODH acetylation. (A‐F) HK‐2 cells were treated with MitoQ (5 µmol L^−1^) for 1 h before cisplatin (10 µmol L^−1^). HK‐2 cells were collected at 72 h after cisplatin. Mito Sox was used to evaluate mitochondrial ROS by flow cytometry. The experiment was repeated two times. N = 3. (A) Representative pictures. (B) Mitochondrial ROS. (C‐F) Co‐IP was used to analyze SIRT3 SUMOylation in preteated‐HK‐2 cells. The experiment was repeated two times. N = 3. (C) Representative pictures. (D) SUMOylated SIRT3. (E) SIRT3. (F) DHODH. G‐T) Adult ICR male mice were exposed with cisplatin (20 mg kg^−1^, intraperitoneal injection). MitoQ (5 mg kg^−1^) were pretreated 24 h before cisplatin. Mouse kidneys were collected 72 h after cisplatin. (G‐K) Western blot was used to analyze four subunits of oxidative phosphorylation. The experiment was repeated two times. N = 3. (G) Representative pictures. (H) CV‐ATP5A. (I) CIII‐UQCRC2. (J) CII‐SDHB. (K) CI‐NDUFB8. (L‐N) Renal SOD2 and IDH2 were analyzed by Western blot. (L) Representative pictures. The experiment was repeated two times. N = 3. (M) SOD2. (N) IDH2. (O‐Q) Co‐IP was used to analyze SIRT3 SUMOylation. The experiment was repeated two times. N = 3. (O) Representative pictures. (P) SUMOylated SIRT3. (Q) SIRT3. (R‐T) Co‐IP was used to analyze DHODH Acetylation. The experiment was repeated two times. N = 3. (R) Representative pictures. (S) Acetylated DHODH. (T) DHODH. Quantitative data were shown as data pots and S.E.M. *p < 0.05, **p < 0.01.

It is accepted that MitoQ, a well‐known mitochondria‐target antioxidant, could improve mitochondrial function.^[^ ^35^, ^36^ ^]^ To determine the role of mitochondrial ROS in cisplatin‐induced SIRT3 SUMOylation, HK‐2 cells were pretreated with MitoQ. As expected, cisplatin‐induced mitochondrial ROS were suppressed in MitoQ‐pretreated HK‐2 cells (**Figure** **8**A,B). Cisplatin‐induced SIRT3 SUMOylation was attenuated in MitoQ‐pretreated HK‐2 cells (Figure 8C,D). Cisplatin‐induced reduction of SIRT3 was alleviated in MitoQ‐pretreated HK‐2 cells (Figure 8C,E). Accordingly, cisplatin‐induced reduction of DHODH was alleviated in MitoQ‐pretreated HK‐2 cells (Figure 8C,F). To further verify the role of mitochondrial dysfunction in cisplatin‐induced SIRT3 SUMOylation, mice were pretreated with MitoQ. As shown in Figure 8G–K, cisplatin‐induced downregulation of renal CV‐ATP5A, CIII‐UQCRC2, and CII‐SDHB were alleviated in MitoQ‐pretreated mice. Moreover, cisplatin‐induced reduction of renal SOD2 and IDH2 was attenuated in MitoQ‐pretreated mice (Figure 8L–N). Next, the effect of pretreatment with MitoQ on cisplatin‐induced renal SIRT3 SUMOylation was evaluated in mice. As shown in Figure 8O,P, cisplatin‐induced renal SIRT3 SUMOylation was attenuated in MitoQ‐pretreated mice. Cisplatin‐induced renal SIRT3 reduction was alleviated in MitoQ‐pretreated mice (Figure 8O,Q). Finally, the effect of MitoQ pretreatment on cisplatin‐evoked DHODH acetylation was evaluated. As shown in Figure 8R,S, cisplatin‐induced renal DHODH acetylation was attenuated in MitoQ‐pretreated mice. Cisplatin‐induced renal DHODH downregulation was alleviated in MitoQ‐pretreated mice (Figure 8R,T).

### 2.9 MitoQ Protects against Cisplatin‐Evoked Renal Cell Ferroptosis and AKI

**Figure 9:**
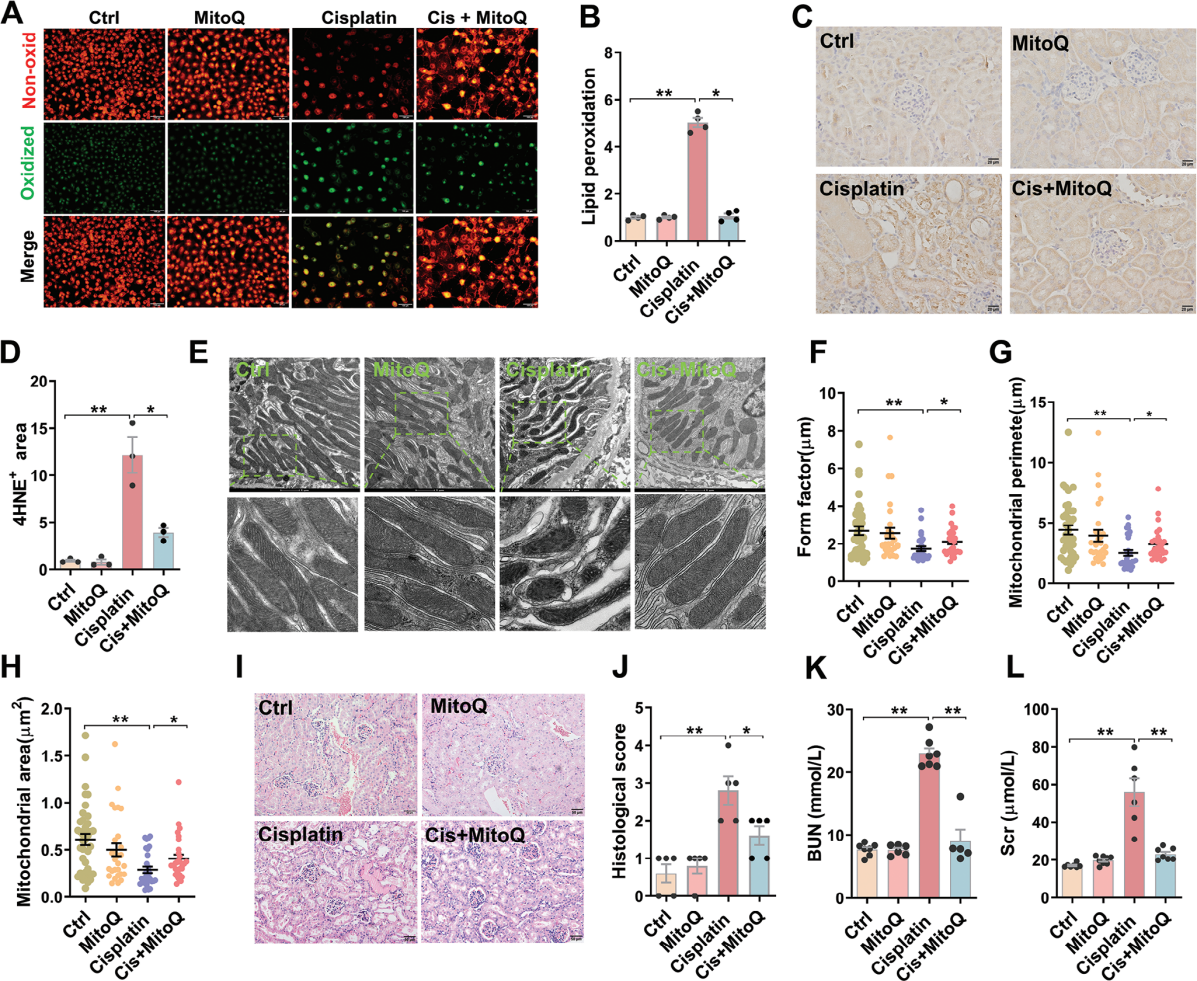
The effect of MitoQ on cisplatin‐induced AKI and renal cell ferroptosis. A‐B HK‐2 cells were treated with MitoQ (5 µmol L^−1^) 1 h before cisplatin (10 µmol L^−1^). HK‐2 cells were collected at 72 h after cisplatin. C11‐BODIPY^581/591^ was used to evaluate lipid peroxidation. The experiment was repeated two times. N = 4. (A) Representative pictures: scale bar = 20 µm. (B) Quantitative analysis of oxidized lipids. C‐L Adult ICR male mice were exposed with cisplatin (20 mg kg^−1^, intraperitoneal injection). MitoQ (5 mg kg^−1^) was pretreated 24 h before cisplatin. Mouse kidneys and blood serum were collected at 72 h after cisplatin. (C‐D) IHC was used to analyze renal 4‐HNE. The experiment was repeated two times. N = 3. (C) Representative pictures: scale bar = 20 µm. (D) Renal 4‐HNE^+^ area. E‐H) Mitochondrial ultrastructure of renal cells was observed using TEM. (E) Representative pictures. (F) Form factor. (G) Mitochondrial perimeter. (H) Mitochondrial area. I‐J) H&E staining was used to evaluate renal histopathology. N = 5 (I) Representative pictures: scale bar = 50 µm. (J) Histopathological scores. K‐L) Renal function was measured. N = 5‐7. (K) Scr. (L) BUN. Quantitative data were shown as data pots and S.E.M. *p < 0.05, **p < 0.01.

The effect of MitoQ on cisplatin‐evoked renal cell ferroptosis was analyzed. As expected, cisplatin‐evoked lipid peroxidation, as evaluated using immunofluorescence, was attenuated by MitoQ supplement (**Figure** **9**A,B). Moreover, cisplatin‐evoked elevation of renal 4‐HNE^+^ area was reversed by MitoQ supplement (Figure 9C,D). Besides, cisplatin‐induced mitochondrial morphological changes were alleviated in MitoQ‐pretreated mouse kidneys (Figure 9E–H). Finally, cisplatin‐induced renal dysfunction and pathological damage were improved in MitoQ‐pretreated mouse kidneys (Figure 9I–L).

## 3 Discussion

Several studies confirmed that characteristic indices of renal cell ferroptosis, such as renal MDA, 4‐HNE, and oxidant lipid contents, were upregulated during cisplatin‐evoked AKI.^[^ ^7^, ^37^, ^38^ ^]^ In this study, RNA‐seq analysis showed that ferroptosis pathway was enriched in cisplatin‐treated HK‐2 cells. Moreover, oxidant lipids were elevated in cisplatin‐treated mouse kidneys and HK‐2 cells. In addition, Fer‐1 protected mice from cisplatin‐induced AKI. It is accepted that oxidized AA metabolites are the major oxidized lipids for ferroptosis.^[^ ^39^ ^]^ In this study, we showed that the contents of oxidized AA metabolites, such as 12‐oxoETE, 14,15‐ETE, 5HETE, PGA2, and 5,6‐ETE, were increased in cisplatin‐treated HK‐2 cells. Moreover, oxidized metabolites of other unsaturated fatty acids, such as DHA, GLA, EPA, and LA, were elevated in cisplatin‐treated HK‐2 cells. These results provide novel data that renal cell ferroptosis is involved in cisplatin‐evoked AKI.

It is well accepted that GPX4 is a key regulator for ferroptosis.^[^ ^21^ ^]^ However, a recent study indicated that GPX4 did not play a key role in cisplatin‐induced renal cell ferroptosis.^[^ ^7^ ^]^ To explore the potential mechanism of cisplatin‐induced renal cell ferroptosis, targeted metabolomics was used to analyze cellular metabolites in cisplatin‐treated HK‐2 cells. We showed that pyrimidine biosynthesis was disrupted, during which UMP, an end product of pyrimidine biosynthesis, was reduced and ASP, an initiating substrate of pyrimidine biosynthesis, was elevated in cisplatin‐treated HK‐2 cells. DHODH, located in mitochondrial inner membrane, is the critical enzyme for de novo pyrimidine synthesis.^[^ ^40^ ^]^ A recent study has demonstrated that DHODH is a novel ferroptosis defense mechanism.^[^ ^26^, ^27^ ^]^ In this study, our results showed that renal mitochondrial DHODH was reduced in cisplatin‐treated mice. It is well known that DHODH could convert CoQ to CoQH_2_ in de novo pyrimidine synthesis.^[^ ^41^ ^]^ In our study, CoQH_2_ content was decreased in cisplatin‐treated HK‐2 cells. By contrast, CoQ content was increased in cisplatin‐treated HK‐2 cells. To explore the potential role of DHODH reduction in cisplatin‐evoked renal cell ferroptosis, HK‐2 cells were pretreated with either DHODH plasmids or siRNA‐targeted DHODH. Cisplatin‐induced cellular CoQH_2_ depletion was attenuated in DHODH*^OE^* HK‐2 cells. Correspondingly, cisplatin‐induced elevation of oxidized lipid metabolites was alleviated in DHODH*^OE^* HK‐2 cells. By contrast, cisplatin‐induced cellular CoQH_2_ depletion was aggravated and cisplatin‐caused elevation of oxidized lipid metabolites was exacerbated in DHODH‐silenced HK‐2 cells. Taken together, these results suggest that mitochondrial DHODH reduction is partially involved in cisplatin‐evoked renal cell ferroptosis.

Increasing evidences demonstrate that acetylation and deacetylation are important post‐translational modifications of mitochondrial proteins.^[^ ^42^ ^]^ Numerous studies have confirmed that acetylation modification promotes degradation of mitochondrial proteins.^[^ ^43^ ^]^ DHODH is located in mitochondrial intermembrane. The present study found that acetylated DHODH was elevated in cisplatin‐treated mouse kidneys and HK‐2 cells. By contrast, mitochondrial DHODH protein was reduced in cisplatin‐treated mouse kidneys and HK‐2 cells. Thus, we guess that cisplatin reduces DHODH protein by promoting mitochondrial DHODH acetylation. SIRT3, SIRT4, and SIRT5, localized in mitochondrial inner membrane, function as NAD^+^‐dependent deacetylases that inhibit acetylation of mitochondrial proteins.^[^ ^44^ ^]^ In this study, no difference on renal SIRT4 was observed between cisplatin‐treated mice and controls. Moreover, renal SIRT5 was only slightly reduced in cisplatin‐treated mice. Interestingly, mitochondrial SIRT3 was obviously decreased in cisplatin‐treated HK‐2 cells and mouse kidneys. To determine the possible role of SIRT3 reduction on cisplatin‐induced DHODH acetylation, HK‐2 cells were overexpressed with SIRT3. We found that cisplatin‐induced DHODH acetylation was alleviated in SIRT3*^OE^* HK‐2 cells. Correspondingly, cisplatin‐induced DHODH reduction was reversed in SIRT3*^OE^* HK‐2 cells. Moreover, cisplatin‐induced renal lipid peroxidation was alleviated in SIRT3*^OE^* HK‐2 cells. NMN is a precursor of NAD^+^.^[^ ^45^, ^46^ ^]^ In this study, mice were pretreated with NMN to activate mitochondrial SIRT3 activity. Our results showed that cisplatin‐induced mitochondrial DHODH acetylation was attenuated in NMN‐pretreated mice. Correspondingly, cisplatin‐induced DHODH reduction was reversed by NMN. Moreover, cisplatin‐induced renal lipid peroxidation was alleviated in NMN‐pretreated mice. Cisplatin‐induced AKI was improved by NMN pretreatment. To further verify the role of SIRT3 reduction on cisplatin‐induced DHODH acetylation, *Sirt3* ^−/−^ mice were constructed. As expected, renal acetylated DHODH was elevated in *Sirt3* ^−/−^ mice as compared with wide‐type mice. Interestingly, cisplatin‐induced renal mitochondrial DHODH acetylation was aggravated in *Sirt3* ^−/−^ mice, which could not be reversed by NMN supplementation. Moreover, cisplatin‐induced renal lipid peroxidation was aggravated in *Sirt3^−/‐^* mice, which could also not be rescued by NMN supplementation. In addition, cisplatin‐induced AKI was more serious in *Sirt3^−/‐^* mice, which could not be alleviated by NMN supplementation. Taken together, these results suggest that SIRT3 reduction may partially contribute to cisplatin‐induced mitochondrial DHODH acetylation and renal cell ferroptosis.

SUMOylation is a major modification for SIRT3.^[^ ^47^ ^]^ In this study, we found that mitochondrial function, including oxygen consumption rate (OCR) and extracellular acidification rate (ECAR), was impaired in cisplatin‐exposed HK‐2 cells. Moreover, mitochondrial membrane potential was decreased in cisplatin‐treated HK‐2 cells. Further analysis found that mitochondrial ROS was increased in cisplatin‐treated HK‐2 cells. SIRT3 SUMOylation was accordingly elevated in cisplatin‐treated HK‐2 cells and mouse kidneys. Accumulating data have demonstrated that ROS are the key molecules regulating SUMOylation modification of cellular proteins.^[^ ^48^ ^]^ On the other hand, SIRT3 SUMOylation could reversely downregulate mitochondrial function and elevate ROS production through inhibiting mitochondrial SIRT3 activity.^[^ ^32^, ^33^, ^37^ ^]^ To determine the causal relationship between mitochondrial ROS and SIRT3 SUMOylation, the effect of pretreatment with MitoQ, a mitochondria‐targeted antioxidant, on cisplatin‐induced SIRT3 SUMOylation was evaluated. As expected, cisplatin‐evoked mitochondrial ROS was suppressed in MitoQ‐pretreated HK‐2 cells. Interestingly, cisplatin‐induced SIRT3 SUMOylation was attenuated in MitoQ‐pretreated HK‐2 cells and mouse kidneys. Moreover, cisplatin‐induced renal DHODH acetylation was attenuated in MitoQ‐pretreated mice. In addition, cisplatin‐caused renal cell ferroptosis and AKI were alleviated in MitoQ‐pretreated mice. These results provide partial evidences that mitochondrial ROS mediate cisplatin‐induced SIRT3 SUMOylation and subsequent DHODH acetylation.

It is widely accepted that mitochondrial SIRT3 activity was reduced in senescent cells, including aged‐related senescence and stress‐evoked cellular senescence.^[^ ^49^ ^]^ Indeed, SIRT3 is an NAD^+^‐dependent deacetylase. Accumulating data demonstrate that supplementation with NAD^+^ precursor could alleviate stress‐evoked mitochondrial dysfunction and organ damage, including PM2.5‐induced pulmonary fibrosis, ischemia‐reperfusion‐induced AKI and sepsis‐induced acute lung injury.^[^ ^50^, ^51^, ^52^ ^]^ Several studies have explored the effect of NAD^+^ precursor on age‐related senescence with negative results.^[^ ^50^ ^]^ An earlier study found that NMN supplementation could not improve age‐related mitochondrial dysfunction and grip strength decline in elderly men.^[^ ^49^, ^53^ ^]^ In the present study, we found that pretreatment with NMN protected mice from cisplatin‐induced renal cell ferroptosis and AKI in wild‐type mice. Interestingly, supplement with NMN could not prevent cisplatin‐induced renal cell ferroptosis and AKI in *Sirt3* ^−/−^ mice. Our results provide novel evidence that NAD^+^ precursor could be used to alleviate stress‐evoked mitochondrial dysfunction and organ damage through improving mitochondrial SIRT3 activity.

The current results confirmed that SIRT3 SUMOylation‐mediated mitochondrial DHODH acetylation partially contributes to cisplatin‐induced renal cell ferroptosis. The current study has several limitations. First, the current study did not use DHODH genetic mice or viral interventions to explore the role of DHODH on cisplatin‐induced renal cell ferroptosis. Secondly, the current study did not use SIRT3 conditional knockout mice, such as renal tubule‐specific knockout models, to explore the role of SIRT3 on cisplatin‐induced DHODH acetylation and renal cell ferroptosis. Finally, the present study just considered the role of mitochondrial deacetylases on cisplatin‐induced mitochondrial DHODH acetylation. A recent study indicated that mitochondrial acetylase HADC3 inhibited hydroxyacyl‐CoA dehydrogenase (HADAH) activity by promoting its acetylation.^[^ ^54^ ^]^ Thus, additional work is required to further explore the role of mitochondrial acetylases on cisplatin‐induced DHODH acetylation and subsequent renal cell ferroptosis.

In summary, the present study investigated the role of renal cell ferroptosis in cisplatin‐induced AKI. Oxidized arachidonic acid metabolites, determined by targeted oxidized lipid metabolomics, were elevated in cisplatin‐treated renal cells. Targeted metabolomics showed that pyrimidine biosynthesis was disrupted in cisplatin‐treated mouse kidneys. The reduction of mitochondrial DHODH, a critical enzyme during pyrimidine synthesis, was involved in cisplatin‐induced renal cell ferroptosis. We found that mitochondrial SIRT3 was decreased in cisplatin‐exposed mouse kidneys. Our results provide evidence that mitochondrial DHODH acetylation, mediated by SIRT3 SUMOylation, partially contributes to cisplatin‐induced renal cell ferroptosis. Thus, supplementation with MitoQ, a mitochondria‐targeted antioxidant, and NMN, a precursor for NAD^+^, could efficiently prevent cisplatin‐induced renal cell ferroptosis and AKI.

## 4 Experimental Section

### Animals and Treatments

Adult ICR male mice, adult male wide type C57BL/6J mice, and C57BL/6J *Sirt3^−/‐^* mice were provided by GemPharmatech Co. (Nanjing, China). Mice were fed in a Specific Pathogen Free Laboratory animal center with a temperature of 20 – 25 °C, humidity of 50 ± 5%, and enough food and water. This study consists of five independent experiments. Experiment 1, to evaluate cisplatin‐induced renal cell ferroptosis, adult ICR male mice were intraperitoneally injected with cisplatin (20 mg kg^−1^ dissolved in saline). Blood serum and kidney tissues were collected either 24 or 72 h after cisplatin. Experiment 2, to explore the effect of Fer‐1 on cisplatin‐induced AKI, 32 C57BL/6J adult male mice were divided into four groups: Ctrl, Fer‐1 (5 mg kg^−1^), Cis and Cis+Fer‐1 groups. In the Cis and Cis+Fer‐1 groups, mice were injected with a single dose of cisplatin (20 mg kg^−1^). In the Fer‐1 and Cis+Fer‐1 groups, mice were injected with Fer‐1 (5 mg kg^−1^) before cisplatin injection. The dose of Fer‐1 was referred to in our previous study.^[^ ^55^ ^]^ All mice were sacrificed at 72 h after cisplatin. Kidney tissues and blood serum were collected. Experiment 3, to investigate the protective effect of NMN on cisplatin‐induced renal cell ferroptosis, a total of 32 adult male ICR mice were divided into four groups: Ctrl, NMN (500 mg kg^−1^ dissolved in saline), Cis (20 mg kg^−1^ dissolved in saline) and Cis+NMN. Mice were daily pretreated with NMN for consecutive five days and then intraperitoneally injected with cisplatin once. The dose of NMN in this study referred to others.^[^ ^56^ ^]^ All mice were sacrificed at 72 h after cisplatin. Kidney tissues and blood serum were collected. Experiment 4, to investigate the role of Sirt3 on cisplatin‐induced renal cell ferroptosis, 32 wide type and 32 C57BL/6J *Sirt3^−/−^* adult male mice were divided into eight groups: WT, WT+NMN (500 mg kg^−1^), WT+Cis (20 mg kg^−1^), WT+NMN+Cis, *Sirt3^−/−^*, *Sirt3^−/−^* +NMN, *Sirt3^−/−^* +Cis and *Sirt3^−/−^* +NMN+Cis groups. Cisplatin was intraperitoneally injected once. Mice were daily pretreated with NMN for consecutive five days. All of mice were sacrificed at 72 h after cisplatin. Blood serum and kidney tissues were collected. Experiment 5, to investigate the role of mitochondrial dysfunction on cisplatin‐induced renal cell ferroptosis, 32 adult ICR male mice were divided into four groups: Ctrl, MitoQ (5 mg kg^−1^), Cis (20 mg kg^−1^) and Cis+MitoQ groups. Cisplatin was intraperitoneally injected once. Some mice were pretreated with MitoQ 24 h before cisplatin. The dose of MitoQ was referred to in our previous study.^[^ ^55^ ^]^ Blood serum and kidney tissues were collected at 72 h after cisplatin. All animal experiments followed the guidelines for humane treatment set by the Association of Laboratory Animal Sciences and the Center for Laboratory Animal Sciences at Anhui Medical University (approval number: LLSC20220397).

### Chemicals and Reagents

Cisplatin (No: P4394) was bought from Sigma‐Aldrich (St. Louis, MO, USA). Nicotinamide mononucleotide (NMN, No: 1094‐61‐7) was bought from Merck (Billerica, MA, USA). Mitoquinone (MitoQ, ≥98.45% purity, No: 91753‐05‐0) and Ferrostatin‐1 (Fer‐1, No: 347174‐05‐4) was obtained from MedChem Express (New Jersey, USA). Human ubiquinone (CoQ) and human ubiquinol (CoQH_2_) ELISA kits were bought from Kunshan Yuanmu Biotechnology Co., Ltd (Suzhou, China). BODIPY 581/591 C11(No: D3861), Lipofectamine 3000 (No: L3000008), and Mito SOX Red kits (No: M22425) were purchased from Thermo Fisher Scientific (Shanghai, China). Human short‐interfering RNAs, plasmids targeting DHODH, and plasmids targeting SIRT3 were purchased from GenePharma (Shanghai, China). Renal function detection kits were bought from Erkn Biological technology CO., Ltd (Wenzhou, China). Mitochondrial membrane potential assay kit with JC‐1 (No: C2003S) was purchased from Beyotime Biotechnology (Shanghai, China). Seahorse XF Cell Mito Stress Test Kit (No: 103010–100), XF calibrant (No:103 059), XF 100 mM pyruvate solution (No:103578‐100), XF 1.0m Glucose solution (No:103577‐100), XF DMEM (No:103 575), 200 mM glutamine (No:103 579), cell culture miniplate and Extracellular Flux Cartridge were bought from Agilent Seahorse (California, USA). Anti‐4‐hydroxynonenal (4‐HNE, No: ab46545), anti‐SIRT3 (No: ab246522), anti‐DHODH (No: ab174288), anti‐SOD2 (No: ab68155), anti‐DRP1 (No: ab184247), anti‐GPX4 (No: ab125066), anti‐SUMO1 (No: ab32058), anti‐OXPHOS (No: ab110411) and anti‐IDH2 (No: ab131263) antibodies were purchased from Abcam (Cambridge, MA, USA). IP detection reagents (No: ab131366) were purchased from Abcam (Cambridge, MA, USA). Anti‐acetylated‐lysine (No: 9441), anti‐SIRT3 (No: D22A3), SIRT5 (No: 8782) and anti‐TOM20 (No: 42 406) were provided by Cell Signaling Technology (Danvers, MA, USA). Anti‐SIRT4 (No:PAB35354) was bought from Bioswamp (Wuhan, China). Anti‐ACTIN antibody (No: BS6007M) was bought from Bioworld `Technology Ltd (Dublin, Ohio). Anti‐rabbit/mouse HRP‐Streptavidin antibody was bought from BioSharp (Hefei, China). The secondary antibody conjugated with Alexa Fluor 488 and Cy3TM was purchased from Beyotime Biotechnology (Shanghai, China). Deuterated internal standards and eicosanoids were bought from Cayman Chemical (Michigan, USA). Acetonitrile of HPLC‐grade (CAN) and methanol (MeOH) were bought from Merck (Darmstadt, Germany). MilliQ water was bought from Millipore (Bradford, USA). Acetic acid was purchased from Sigma‐Aldrich (St. Louis, MO, USA). CNW Poly‐Sery MAX SPE cartridges were bought from AN PEL Co. (Shanghai, PRC).

### Identification of Knockout Mouse

Briefly, 0.5 cm mouse tail was digested in 500 µL tail digestion buffer overnight at 55 °C, then centrifuged at 12 000 rpm at 4 °C for 1 min. Collected supernatant 300 µL, added anhydrous ethanol 600 µL and gently shaken until white flocculence coming. The precipitate was collected at 12 000 rpm at 4 °C for 15 min, then purged DNA with 70% ethanol and repeated two times. DNA was dissolved in deionized water and genotyped by agarose gel electrophoresis.

### Mitochondrial Isolation

Mitochondria were isolated from fresh renal tissues according to differential centrifugation as described.^[^ ^57^ ^]^ Fresh renal tissues were harvested without freezing. Tissues were incubated in ice‐cold HIM buffer. Then tissues were further homogenized in HIM buffer with a tight Dounce Tissue Grinder 15 times. The debris in homogenates was removed using centrifugation at 800 rpm. The supernatant was centrifugated at 12 000 rpm for 10 min to separate mitochondria and cytoplasm. The sediment containing mitochondrial fraction was re‐suspended using HIM buffer.

### Biochemical Analysis

Mouse blood serum was collected. Renal function indexes (Scr and BUN) were evaluated using kits accordance with manufacturers.

### Histopathology and Immunohistochemistry

Fresh renal tissues were fixed with 4% formaldehyde, and dehydrated. Renal tissues were embedded in paraffin and sectioned at a thickness of 5 – 10 µm. After that, sections were incubated with hematoxylin and eosin staining kits. Immunohistochemistry (IHC) of DHODH and 4‐HNE was evaluated as described.

### Immunofluorescence Staining

Mouse kidneys were embedded in tissue‐freezing medium (SAKURA Tissue‐Tek O.C.T. Compound 4583). After that, nitrogen was used to snap‐frozen samples. Then samples were cut into 5 – 10 µm sections. Cisplatin‐treated HK‐2 cells were fixed, permeabilized, and blocked at 37 °C. Next, tissue sections or cells were incubated with the indicated primary antibody overnight at 4 °C, followed by a secondary antibody for 2 h at 37 °C. Finally, sections were observed by A Leica Thunder confocal microscopy.

### Coimmunoprecipitation

Immunoprecipitation of DHODH, SOD2, and ATP5A were performed with DHODH to confirm the level of acetylated DHODH, SOD2 and ATP5A. Immunoprecipitation of Acetylated‐Lysine was performed with DHODH to evaluate the level of the level of acetylated DHODH. Immunoprecipitation SIRT3 was performed with SUMO1 to evaluate the level of SUMOylated SIRT3. All renal tissue lysates were treated with primary antibody overnight at 4 °C.

### Mitochondrial Ultrastructure

Fresh renal tissues were cut into 1 mm^3^. Then, renal samples were fixed in 2.5% glutaraldehyde at 4 °C. Renal samples were embedded and cut into 70–100 nm sections by Leica EMUC7. Sections were incubated with uranium lead double to stain. Transmission electron microscopy (TEM, Thermoscientific Talos L120C G2) was used to observe mitochondrial ultrastructure.

### Cell Culture

HK‐2 (human kidney) cell, a proximal tubular cell (PTC) line, were incubated in a DMEM/F12 (Thermo Fisher Scientific), 10% fetal bovine serum (Biological Industries), and 1% 100X Penicillin‐Streptomycin Solution (Beyotime Biotechnology). HK‐2 cells were cultured in an incubator with humidity (95%), carbon dioxide (5%), and temperature (37 °C).

### Gene Silence and Overexpression

The in vitro DHODH silence was achieved by siRNA, purchased from GenePharma (Shanghai, China). The DHODH and SIRT3 overexpression plasmid were purchased from GenePharma (Shanghai, China). The p DNA3.1 vector was used for plasmid construction. DHODH siRNA and plasmid were transferred into HK‐2 cells using Lipofectamine 2000. The effect of knockdown and overexpression was confirmed 24 h after transfection.

### RNA Sequencing

Total RNA was extracted using Trizol Reagent (Invitrogen Life Technologies). concentration, quality, and integrity of RNA were detected using a NanoDrop spectrophotometer (Thermo Scientific). Three micrograms of RNA acted as input material. Then, sequencing libraries were generated. The sequencing library was sequenced on the NovaSeq 6000 platform (Illumina) Shanghai Personal Biotechnology Cp. Ltd. GO and pathway enrichment were analyzed by https://www.bioi nformatics.com.cn (last accessed on 10 Nov 2023). Gene set enrichment analyses were conducted using GSEA (version 4.3.2). Mitochondrial genes were downloaded from MitoCarta database (version 3.0).^[^ ^58^ ^]^

### Mitochondrial ROS Measurement

Mitochondrial ROS was evaluated using Mito Sox. HK‐2 cells cultured until 80% confluency in 24 wells with three parallel wells. Then, suspended and centrifuged at 800 rpm for 5 min. Removed supernatant and added 200 µL Mito Sox (5 µmol L^−1^) fluorescence probe for 20 min at 37 °C. Finally, flow cytometry (Beckman, USA) was used to detect the level of mitochondrial ROS.

### Seahorse Cell‐Mito Stress Tests

OCR, and ECAR were analyzed by Seahorse XF Cell‐Mito Stress tests. First, HK‐2 cells were seeded in XFp cell culture microplates cell culture miniplate 8000 cells per well. Then putted XF Calibrant 5 mL and hydrated extracellular flux cartridge in 37 °C CO_2_‐free incubator overnight. Next day, XFp cell culture microplates were incubated with a preheated test medium for 1 h. After that, FCCP concentration was verified through FCCP test. After cisplatin exposure, the FCCP (1.5 µM), the Oligomycin (1.5 µM) and Rotenone/Antimycin A (0.5 µM) were loaded into the port of the probe plate. Finally, the s Seahorse XF Cell‐Mito Stress tests was conducted on the Seahorse XFp Extracellular Flux Analyzer (Agilent).

### Mitochondrial Membrane Potential Assay

Mitochondrial membrane potential was evaluated using mitochondrial membrane potential assay kit with JC‐1. HK‐2 cells cultured until 80% confluency in 24 wells with three parallel wells. JC‐1 dye (10 µM L^−1^) incubated for 25 min at 37 °C. Then cells were washed using warm PBS. Finally, cells were observed using Leica Thunder confocal microscopy.

### Oxidized lipids Measurement

Oxidized lipids were detected using C11‐BODIPY^581/591^ dye. HK‐2 cells cultured until 80% confluency in 24 wells with three parallel wells. C11‐BODIPY^581/591^ dye (10 µM L^−1^) incubated for 25 min at 37 °C. Then cells were washed using warm PBS. Finally, cells were observed using Leica Thunder confocal microscopy.

### Quantification of Cellular Metabolites by LC‐MS/MS

Renal tissues were placed on ice to thaw. 100 µL ultrapure water extract was added to resuspend the cell. Divided 50 µL cell suspension and added methanol (200 µL precooled at −20 °C), then vortexed for 2 min. Renal samples were frozen in nitrogen for 6 min, placed on ice for 6 min to thaw, and then vortexed for 3 min. The above step was repeated 3 times. Renal samples were centrifuged at 12 000 rpm at 4 °C for 12 min. 400 µL supernatant was transferred, placed in refrigerator at −20 °C for 25 min, and centrifuged at 12 000 rpm at 4 °C for 15 min. After that, 180 µL supernatant was transferred through the Protein Precipitation Plate. The left cell suspension was used to determine the protein concentration. Renal sample extracts were analyzed by LC‐ESI‐MS/MS system (Waters ACQUITY H‐Class, http://www.waters.com/nextgen/us/en.html; MS, QTRAP 6500+ System, http://sciex.com/).

### Quantification of Oxidized Lipids by LC‐MS/MS

Cisplatin‐exposed HK‐2 cells were harvested at 72 h. The cells were quickly washed using PBS. HK‐2 cells were suspended. After that, suspension cells were centrifuged at 800 rpm at 4 °C for 10 min and removed supernatant. 100 µL of ultrapure water was added to resuspend the cell. Divided 50 µL cell suspension and added 200 µL methanol/acetonitrile (1:1, v/v) solution containing internal standard. Samples were vortexed for 10 min and then placed on ice to thaw. The above freeze‐thaw step was repeated four times. Samples were placed at −20 °C for 30 min to precipitate protein. Then, centrifuged at 12 000 rpm at 4 °C for 12 min, collected supernatant, and transferred. The left 50 µL cell suspension was taken to evaluate protein concentration. Samples extracts were analyzed using an LC‐ESI‐MS/MS system (UPLC, ExionlLC AD, http://sciex.com.cn/; MS, QTRAP®6500+ System, https://sciex.com/).

### Real‐Time qPCR (RT‐PCR)

Renal tissue total RNAs were extracted using Trizol reagent. After RNA quantification, RNase‐Free DNA enzyme digestion, and reverse transcription, cDNA was constructed. The mRNA levels of specific genes were then elevated by a Real‐time fluorescence quantitative PCR machine (Roche, SWIT).

### CoQ and CoQH2 Measurement

HK‐2 cells were treated with cisplatin (10 µmol L^−1^) for 72 h. Briefly, cells were grown in 60 mm plates to ≈80% confluence. The cells were quickly washed in 2 ml PBS (PH = 7.2∼7.4) to remove serum‐derived CoQ and CoQH_2_. Cells were suspended. Then, suspension cells were centrifuged at 800 rpm for 20 min at 4 °C. Removed supernatant and cells were diluted in PBS. Repeated freeze‐thaw cycles to release intracellular components. Samples were centrifuged at 3000 rpm for 20 min at 4 °C. Finally, the contents of CoQ and CoQH_2_ were evaluated.

### Western Blotting

Protein of renal tissues and HK‐2 cells were extracted. Then, protein concentrations were quantified and boiled at 100 °C for 10 min. Next, protein samples were separated by SDS‐PAGE, and transferred to a PVDF membrane. The membrane was blocked with fat‐free 5% milk. After incubation with the corresponding primary and secondary antibodies, protein expression was observed by the ChemiDocTouch Imaging System (Bio‐Rad).

### Statistical Analysis

Quantitative data were shown as data pots and S.E.M. ANOVA and Student‐Newmann‐Keuls post hoc method were used to analyze the difference among different groups. Student *t*‐test was used to the comparison between two groups. *P*<0.05 was indicated as statistically significant.

## Author Contributions

N.‐N.L., Y.‐Y.G., and X.‐Y.Z. contributed equally to this work. N.N.L., D.X.X, and S.X. performed conceptualization. R.Y.H., Z.B.L., and Y.Z.H. performed resources. Y.H.Z. performed methodology. X.Y.Z. and D.X.Y. performed visualization. D.X.X. and S.X. performed supervision. S.X. founds. N.N.L. performed writing—original draft. N.N.L., D.X.X., and S.X performed writing—review & editing.

## Conflict of Interest

The authors declare no conflict of interest.

## Supporting information



Supporting Information

## Data Availability

The data that support the findings of this study are available from the corresponding author upon reasonable request.
