# Supplementary material for: Mitochondrial Dysfunction‐Evoked DHODH Acetylation is Involved in Renal Cell Ferroptosis during Cisplatin‐Induced Acute Kidney Injury
Source: Adv Sci (Weinh). 2024 Sep 20;11(43):2404753. doi: 10.1002/advs.202404753 (PMC11578349; doi:10.1002/advs.202404753)
Supplement: Supplementary file 1 — Supporting Information [file ADVS-11-2404753-s001.docx]

**Supplementary Figure S1**

**Supplementary Figure.1. Cisplatin-induced acute kidney injury and renal cell ferroptosis**. **A**-**J** Adult male CD-1 mice were intraperitoneally injected with cisplatin (20 mg/kg). Kidney and blood serum were collected either 24 or 72 h after cisplatin. (**A**-**B**) Renal histopathology was detected by H&E staining. (**A**) Representative pictures: Scale bar = 50 μm. (**C**) Histopathological scores. **D**, **E** Renal function was measured. (**D**) BUN; (**E**) Scr. **E**, **F** Renal 4-HNE residues were evaluated using IHC. (**E**) Representative pictures: Scale bar = 20 μm. (**F**) Renal 4-HNE^+^ area. **G**-**J**, Mitochondrial ultrastructure. (**G**) Original magnification: Scale bar = 1 μm. (**H**) Quantitative analysis of mitochondrial area. (**I**) Quantitative analysis of form factor. (**J**) Quantitative analysis of mitochondrial perimeter. (**K**) HK-2 cells were cultured with cisplatin (10 µmol/L) for 72 h. Heatmap of RNA-sequencing. Experiments were repeated. (**L**-**O**) Adult C57BL/6J male mice were exposed with cisplatin (20 mg/kg, intraperitoneal injection). Fer-1(5 mg/kg) were pretreated 1 h before cisplatin. Mouse kidneys and blood serum were collected 72 h after cisplatin. (**L**-**M**) Renal histopathology was detected by H&E staining. (**L**) Representative pictures: Scale bar = 50 μm. (**M**) Histopathological scores. **N**, **O** Renal function was measured. (**N**) BUN; (**O**) Scr. Quantitative data were shown as data pots and S.E.M. **P*<0.05, ***P*<0.01.

**Supplementary Figure S2**

**
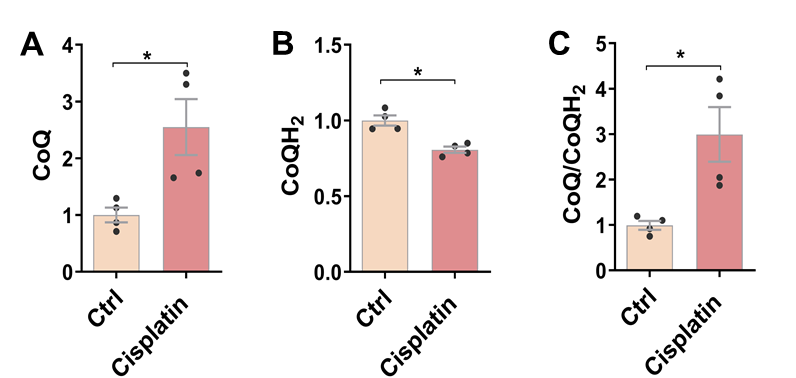
**

**Supplementary Figure 2. The content of CoQH2 and CoQ in cisplatin-treated HK-2 cells** (**A**-**C**). CoQH2 and CoQ were detected in HK-2 cells at 72 h after cisplatin treatment. (**A**) CoQH2. (**B**) CoQ. (**C**) CoQ/CoQH2. Quantitative data were shown as data pots and S.E.M. *P<0.05, **P<0.01.

**Supplementary Figure S3**


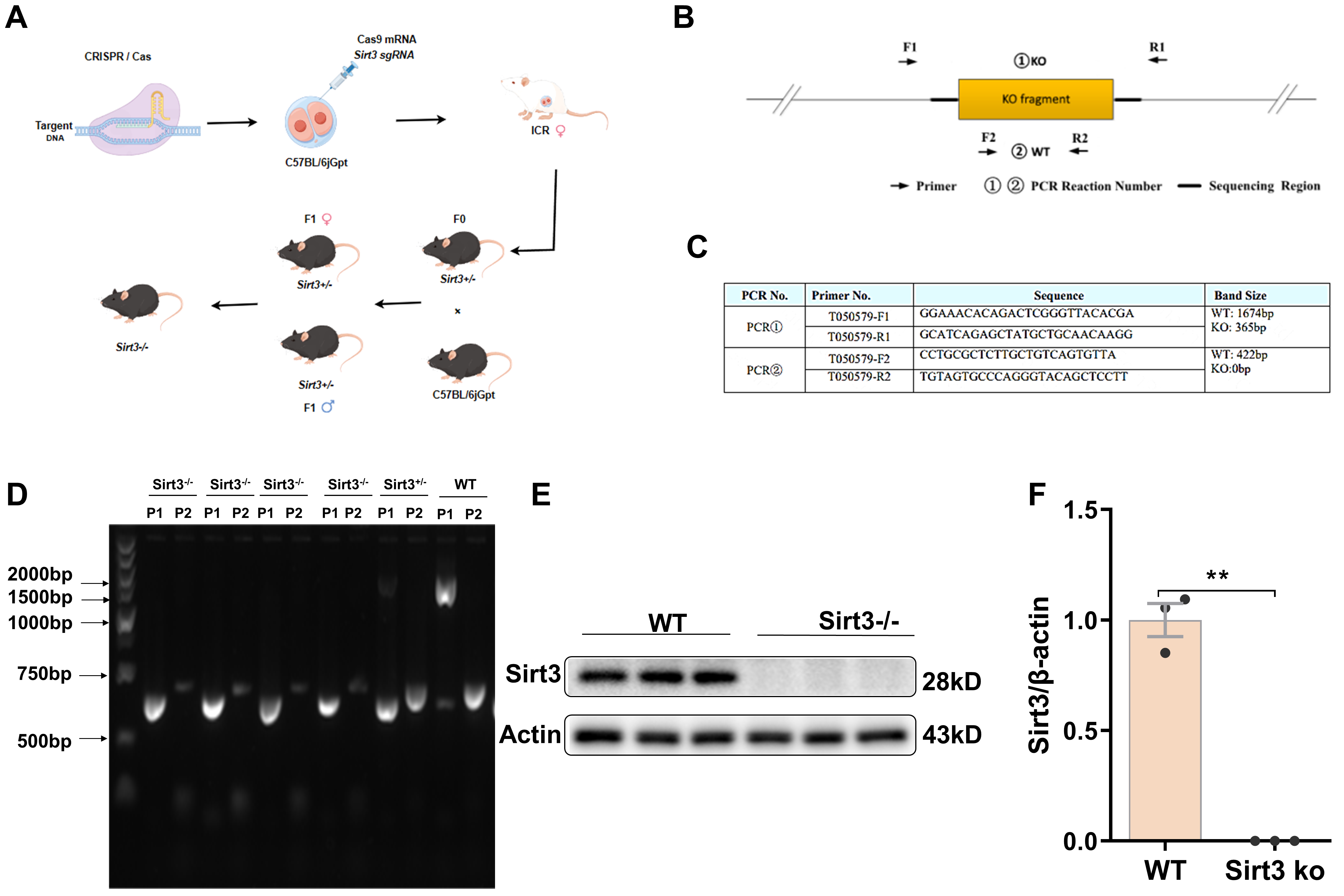


**Supplementary Figure.3. Procedure of Sirt3 knockout construction and identification of genotype.** (**A**) Simplified schematics of *Sirt3* knockout procedure. (**B**) Strategy of Genotyping. (**C**) Primer information. (**D**) Representative pictures of the gel image. (**E**-**F**) Renal SIRT3 was analyzed by Western blot. (**E**) Representative pictures. (**F**) SIRT3. Quantitative data were shown as data pots and S.E.M. **P*<0.05, ***P*<0.01.
